# Treatment with PBI-4050 in patients with Alström syndrome: study protocol for a phase 2, single-Centre, single-arm, open-label trial

**DOI:** 10.1186/s12902-018-0315-6

**Published:** 2018-11-26

**Authors:** Shanat Baig, Vishy Veeranna, Shaun Bolton, Nicola Edwards, Jeremy W. Tomlinson, Konstantinos Manolopoulos, John Moran, Richard P. Steeds, Tarekegn Geberhiwot

**Affiliations:** 1 Centre for Rare Disease, Department of Inherited Metabolic Disorders, Queen Elizabeth Hospital Birmingham, Edgbaston, Birmingham, B15 2TH UK; 2 Institute of Cardiovascular Science, University of Birmingham, Edgbaston, Birmingham, B15 2TT UK; 3 Department of Cardiology, Queen Elizabeth Hospital, Edgbaston, Birmingham, B15 2TH UK; 4 Oxford Centre for Diabetes, Endocrinology & Metabolism, NIHR Oxford Biomedical Research Centre, University of Oxford, Oxford, OX3 7LJ UK; 5 Institute of Metabolism and Systems Research, University of Birmingham, Edgbaston, Birmingham, B15 2TT UK; 6 Prometic Pharma SMT Ltd., Horizon Park, Barton Road, Cambridge, CB23 7AJ UK; 7 Inherited Metabolic Disorders, University Hospital Birmingham NHS Foundation Trust, Mindelsohn Way, Edgbaston, Birmingham, B15 2TH UK

**Keywords:** Alström syndrome, Fibrosis, Insulin resistance, Metabolic syndrome, Diabetes mellitus, Obesity, Cardiovascular, Liver, Magnetic resonance imaging, PBI4050

## Abstract

**Background:**

Alström syndrome (ALMS) is a very rare autosomal recessive monogenic disorder caused by a mutation in the *ALMS1* gene and characterised by childhood onset obesity, dyslipidaemia, advanced non-alcoholic fatty liver disease, diabetes and extreme insulin resistance. There is evidence of multi-organ fibrosis in ALMS and severity of the disease often leads to organ failure with associated morbidities, resulting in reduced life expectancy. There are no specific treatments for this disease, and current management consists of only symptomatic therapies. PBI-4050 is a new molecular entity with demonstrated anti-inflammatory and anti-fibrotic activities in preclinical models, including animal models of human diseases characterized by progressive fibrosis in the kidney, heart, liver and lungs. Moreover, completed Phase 2 studies in type 2 diabetes mellitus with metabolic syndrome and idiopathic pulmonary fibrosis further support the anti-inflammatory and anti-fibrotic activity of PBI-4050. Together, these data suggest that PBI-4050 has the potential to treat the pathological inflammatory and fibrotic features of ALMS. The aim of this study is to evaluate the safety and anti-inflammatory & anti-fibrotic activities of PBI-4050 in subjects with ALMS.

**Methods:**

This is a Phase 2, single-centre, single-arm, open-label trial. A total of 18 patients with ALMS will be enrolled to receive PBI-4050 at a total daily oral dose of 800 mg for an initial 24 weeks with continuation for an additional 36 or 48 weeks. Standard assessments of safety include adverse events, clinical laboratory tests, vital signs, physical examination and electrocardiograms. Efficacy assessments include adipose tissue biopsy, hyperinsulinaemic-euglycaemic glucose clamp, adipose tissue microdialysis, liver transient elastography, liver and cardiac magnetic resonance imaging, and laboratory blood tests.

**Discussion:**

This is the first clinical study of PBI-4050 in subjects with ALMS. Given the rarity and complexity of the disease, a single-centre, single-arm, open-label design has been chosen to maximise subject exposure and increase the likelihood of achieving our study endpoints. The results will provide valuable safety and preliminary evidence of the effects of PBI-4050 in ALMS, a rare heterogeneous disease associated with progressive fibrosis and premature mortality.

**Trial registration:**

The trial is registered on ClinicalTrials.gov (Identifier; NCT02739217, February 2016) and European Union Drug Regulating Authorities Clinical Trials (EudraCT Number 2015–001625-16, Sept 2015).

**Electronic supplementary material:**

The online version of this article (10.1186/s12902-018-0315-6) contains supplementary material, which is available to authorized users.

## Background

Alström syndrome (ALMS) is a rare autosomal recessive genetic disorder with an estimated prevalence of less than one per million [1]. It is characterized by retinal cone-rod dystrophy, hearing loss, childhood obesity, hyperinsulinemia, severe insulin resistance, as well as type 2 diabetes mellitus (T2DM), hypertriglyceridemia and multiple organ dysfunctions due to progressive fibrosis. Increasing systemic fibrosis develops as patients age, with clinical manifestations in multiple organs. The severity of the disease, often leading to organ failure with associated morbidities, results in a reduced life expectancy, rarely exceeding 50 years [1]. There is no specific treatment for this genetic disease and current management consists of symptomatic therapies.

ALMS is caused by mutations in ALMS1, a large gene located on chromosome 2p13 with ubiquitous expression [2, 3]. The ALMS1 protein localizes to the centrosomes and basal bodies of ciliated cells and may play a role in microtubular organization and cilia assembly or function [3, 4]. In addition, this protein has been implicated in intracellular trafficking, regulation of cilia signalling pathways, and cellular differentiation among others [5]. Pathological observations from post-mortem specimens reveal extensive fibrosis in many organs including the kidney, heart, liver, and lung [6]. Furthermore in vitro dermal fibroblast cultures from ALMS patients display cytoskeleton abnormalities, impaired migration, increased production of collagens, have augmented cell cycle length, and show enhanced resistance to apoptosis [7].

PBI-4050 is a potential drug candidate for the treatment of inflammatory and fibrosis-related diseases, including idiopathic pulmonary fibrosis, chronic kidney disease and T2DM with metabolic syndrome. PBI-4050 is a 3-pentylbenzeneacetic acid sodium salt with a molecular weight of 228.3. It is a new molecular entity with demonstrated anti-inflammatory and anti-fibrotic activities in both in vitro and in vivo models. In vitro models have shown that PBI-4050 regulates macrophages, T cells, fibrocytes, fibroblasts, myofibroblasts, and epithelial cells and modulates the over-expression of many pro-inflammatory and pro-fibrotic cytokines and growth factors in different cell types [8–10]. Moreover, when tested against normal human dermal fibroblasts stimulated with transforming growth factor- β, PBI-4050 markedly reduced the production of pro-fibrotic cytokines, including connective tissue growth factor (CTGF) and epidermal growth factor, while restoring the production of anti-fibrotic cytokines such as hepatocyte growth factor [11–14]. PBI-4050 also reduced the production of α-smooth muscle actin, a marker of myofibroblast activity [15]. Pre-clinical testing has shown PBI-4050 to be effective in preventing or reversing fibrosis in all animal models studied, including models of fibrosis in the heart, kidney, lung and liver [12, 13, 16–21]. Furthermore, PBI-4050 has been shown in a spontaneous model of T2DM (the *db*/*db* mouse) to be effective in preventing the loss of insulin production that characterizes the natural history in these animals and leads to death from hyperglycaemia at 24 to 26 weeks of age [22, 23]. Late treatment (from 16 weeks of age) with PBI-4050 restores insulin production and preserves the islet architecture by reducing macrophage and T-cell infiltration [9, 16].

Thus, the available pre-clinical data suggest that PBI-4050 has the potential to treat the pathological inflammatory and fibrotic features of ALMS as outlined in Table 1. Many of these features will be explored in this study as well. The anti-fibrotic effects of PBI-4050 will be assessed by measuring blood and urine biomarkers of fibrosis and inflammation and by performing imaging studies such as transient elastography (TE) or fibroscan of the liver and magnetic resonance imaging (MRI) of the heart and liver. The potential effects on insulin sensitivity will be assessed by standard clinical and research methods, including fasting blood glucose, 4-point glucose profile, hyperinsulinaemic-euglycaemic clamp and adipose tissue microdialysis. The data generated will provide valuable evidence to support further development of PBI-4050 for the treatment of ALMS.

**Table 1:** Major clinical manifestations of Alström syndrome and corresponding pre-clinical effects of PBI-4050

Clinical features of ALMS	Corresponding pre-clinical effects of PBI-4050
Loss of organ function due to fibrosis involving	
Heart	↓ heart fibrosis in suprarenal aortic banding in rats
Lung	↓ lung fibrosis in bleomycin-induced lung fibrosis in mice
Liver	↓ liver fibrosis in CCl4-induced liver fibrosis in rats
Kidneys (renal failure)	↓ kidney fibrosis in various animal models of kidney fibrosis
Type 2 diabetes mellitus	
Early hyperinsulinemia	Reduces insulin resistance in db/db diabetic mice and db/db eNOS−/− diabetic mice
Severe insulin resistance	Normalizes glycaemia in diabetic mice
Late pancreatic failure	Maintains (early treatment) or restores (late treatment) insulin content in pancreatic islets

*eNOS*^*−/−*^ endothelial nitric oxide synthase knockout (mice)

The actual clinical benefits and risks of PBI-4050 are currently unknown in subjects with ALMS, as this is the first study in this subject population. Given the lack of effective treatment for multi-organ fibrosis, a characteristic of ALMS, the proposed study of PBI-4050 is justified based on pre-clinical efficacy data in animal models of fibrosis. Early clinical studies suggest that PBI-4050 is safe and well tolerated after single-dose oral administration at doses ranging from 400 to 2400 mg in healthy volunteers (*n* = 30) and after repeat daily oral doses of 800 mg in subjects with stable renal impairment (*n* = 6), T2DM with metabolic syndrome (*n* = 41), and idiopathic pulmonary fibrosis (*n* = 26), with preliminary evidence of clinical activity in the latter two studies [24, 25]. Across these studies, there have been no deaths but one subject had a study drug-related treatment-emergent serious adverse event (SAE) of pneumonia. Most adverse events (AEs) were mild or moderate in severity, with headache, insomnia, diarrhoea, somnolence, fatigue, and dizziness being the most frequent study drug-related AEs. In addition, there were no clinically significant changes in clinical laboratory, vital signs, or ECG parameters.

### Study objectives

The primary objective is to evaluate the safety and tolerability of 800 mg PBI-4050 (Investigational Medicinal Product [IMP]), administered orally once daily.

The secondary objectives are:

- To evaluate the effect of the IMP on metabolic syndrome parameters
- To evaluate the effect of the IMP on pro-inflammatory/inflammatory, fibrotic, diabetic, and obesity biomarkers in blood and urine
- To evaluate the effect of the IMP on antidiabetic treatment, including oral therapy and insulin requirements

The exploratory objectives are:

- To examine the effect of the IMP on the histological appearances of fat biopsies, in particular the degree of fibrosis and macrophage infiltration;
- To analyse the effect of the IMP on the global metabolome using systemic blood and adipose tissue-specific microdialysis samples;
- To analyse the effect of the IMP on the microdialysate fractions using adipose tissue microdialysis samples;
- To assess the effect of the IMP on liver stiffness using transient elastography;
- To measure the effect of the IMP on the fat content and fibrosis burden in the liver using MRI;
- To measure the effect of the IMP on cardiac fibrosis and function using MRI and on biomarkers, including N-terminal pro-brain natriuretic peptide (NT-pro-BNP);
- To evaluate the effect of the IMP on additional pro-inflammatory/inflammatory, fibrotic, diabetic, and obesity biomarkers in blood and urine;
- To evaluate the effect of the IMP on glucose, insulin, and lipid dynamics using the hyperinsulinaemic-euglycaemic clamp test.

## Methods

**Fig. 1:**
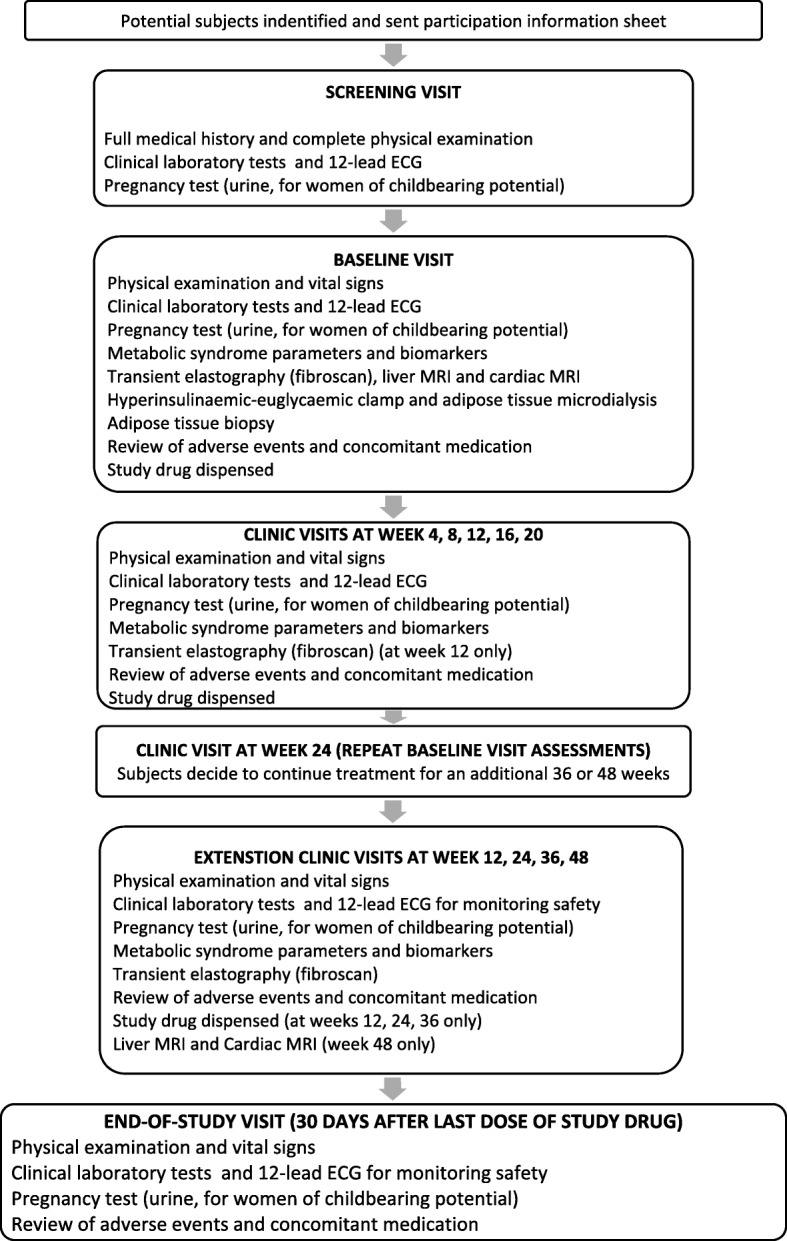
Flowchart of study design

Study PBI-4050-ATX-9-05 is a Phase 2, single-centre, single-arm, open-label study of PBI-4050 in subjects with ALMS. The initial duration of the study is 24 weeks and subjects can elect to enrol into an extension phase of 36 or 48 weeks and subsequently into the ALMS rollover study PBI-4050-CT-9-10. The schedule of study procedures for the enrolment, intervention, and assessments for participants is outlined in Fig. 1 and Table 2 and presented in detail in Additional file 1 (main study) and Additional file 2 (extension period). Subjects will be considered enrolled in the main study at the point when they give written informed consent and treatment with study drug has commenced. Each subject will then be assigned a unique identification number with a country code, site number, and subject number. Subjects may only be included in the 36- or 48-week extension period if informed consent is obtained in advance of any additional study-specific procedures.

**Table 2:** Study visits

Visit	Screen	Wk 1	Wk 4	Wk 8	Wk 12	Wk 16	Wk 20	Wk 24	EP 12	EP 24	EP 36	EP 48	EoS
Informed consent	X							X					
Demography and History	X												
Physical examination	X	X			X			X	X	X	X	X	X
Vital signs	X	X	X	X	X	X	X	X	X	X	X	X	X
Weight & waist	X	X	X	X	X	X	X	X	X	X	X	X	X
Haematology/Biochemistry	X	X	X	X	X	X	X	X	X	X	X	X	X
Urinalysis	X	X	X	X	X	X	X	X	X	X	X	X	X
12-lead ECG	X	X			X			X	X	X	X	X	X
IMP accountability		X	X	X	X	X	X	X	X	X	X	X	
Fasting glucose profile		X	X	X	X	X	X	X	X	X	X	X	X
Adverse events		X	X	X	X	X	X	X	X	X	X	X	X
Concomitant medications		X	X	X	X	X	X	X	X	X	X	X	X
FibroScan		X			X			X	X	X	X	X	
Liver/Cardiac MRI		X						X			X		
Insulin clamp/Microdialysis		X						X					
Adipose tissue biopsy		X						X					
Metabolic syndrome parameter		X			X			X	X	X	X	X	
Biomarkers		X	X	X	X	X	X	X	X	X	X	X	

*Screen* Screening visit, *Wk* weeks, *EP* Extension phase visits

### Location and setting

Study PBI-4050-ATX-9-05 is sponsored by Prometic Biosciences and subjects are recruited from the clinical service at University Hospitals Birmingham NHS Foundation Trust, United Kingdom (UK), which is a nationally commissioned specialist service for ALMS.

### Study population and eligibility criteria

A total of 18 subjects will be enrolled. Subjects should fulfil the following criteria:

- 16 years of age or older at screening;
- A diagnosis of ALMS, confirmed by genetic testing before screening;
- Subjects on diabetes treatment are receiving the same antidiabetic agent for a minimum of 1 month before screening;
- Able and willing to self-monitor blood glucose level with or without assistance;
- Female subjects of childbearing potential must have a negative pregnancy test and agree to use adequate birth control throughout the study and for 30 days after the last dose of study drug;
- Male subjects are willing to use an acceptable contraceptive method throughout the study and for 30 days after the last dose of study drug;
- Able to give written informed consent.

Subjects will not enter the study if any of the exclusion criteria listed in Additional file 3 is fulfilled.

### Intervention

PBI-4050 is administered at a total daily oral dose of 800 mg, 1 h before or 2 h after a meal. Subjects are encouraged to take PBI-4050 consistently at the same time every day. Subjects are prohibited from taking the following concomitant medications:

- Weight loss medications, including natural products;
- Moderate/potent cytochrome P450 (CYP) 2C9 inhibitors (e.g., amiodarone, fluconazole, miconazole, and oxandrolone) and moderate/potent CYP2C9 inducers (e.g., carbamazepine and rifampin);
- Strong CYP3A inhibitors (e.g., ketoconazole, itraconazole, clarithromycin) and strong CYP3A inducers (e.g., rifampin, phenytoin, carbamazepine, and St. John’s wort).

### Study procedure

#### Assessment of safety and tolerability

AEs will be assessed by the investigator starting from screening through 30 days after the last dose of study drug. AEs occurring at the time of the first dose of study drug are referred to as treatment-emergent AEs.

A complete physical examination along with ECGs and vital signs will be performed at each visit. Fasting blood and urine samples will also be collected at each visit for monitoring safety, including haematology, biochemistry, and urinalysis. Clinically significant changes in any of these parameters will be recorded as AEs.

Haematology includes complete blood cell count (CBC), which includes red blood cell count (RBC), haematocrit, and haemoglobin; white blood cell (WBC) count with 5-part differentials (absolute values); and platelet count. Biochemistry includes electrolytes (calcium, sodium, potassium, phosphate, and magnesium), liver function (aspartate aminotransferase [AST], alanine aminotransferase [ALT], gamma-glutamyl transferase [GGT], and total bilirubin), other enzymes and substrates (alkaline phosphatase [ALP]), urea, uric acid, creatinine, albumin, and lactate dehydrogenase, and lipid profile (total cholesterol, HDL-C, low-density lipoprotein cholesterol [LDL-C], triglycerides, total plasma protein).

Urinalysis will include the following macroscopic examinations: pH, specific gravity, protein, glucose, ketones, blood, nitrite, and leukocytes. If the macroscopic shows any abnormal findings, microscopic examination will be performed. Urine pregnancy test will be performed for women with childbearing potential.

Study subjects will be instructed to measure fasting blood glucose each morning and 4-point blood glucose profiles weekly, with the results recorded in a diary. Study personnel will contact the subjects by phone weekly to review the blood glucose results. Subject diaries will be reviewed at each study visit. Hypoglycaemic episodes will be classified as AEs of special interest and defined as follows:

- Suspected symptomatic hypoglycaemia: Symptoms of hypoglycaemia (shaking, dizziness, blurred vision, palpitations, sweating, etc.) with no confirmatory blood glucose level, or blood glucose 3.5–3.9 mmol/L with or without symptoms.
- Confirmed hypoglycaemia: Blood glucose < 3.5 mmol/L with or without symptoms (shaking, dizziness, blurred vision, palpitations, sweating, etc.).
- Severe hypoglycaemia events: Blood glucose < 3.0 mmol/L and/or loss of consciousness.

Details of safety endpoints will be captured on the study electronic case report form.

#### Assessment of metabolic syndrome parameters and biomarkers

Blood samples will be drawn to measure the following metabolic syndrome parameters: Fasting plasma glucose (FPG), Glycated haemoglobin (HbA1c), fasting insulin. HbA1c will be measured at each visit and FPG and fasting insulin will be measured every 12 weeks, as shown in the Schedule of Study Procedures. Homeostasis Model Assessment (HOMA2) for steady state beta cell function (HOMA2-B) and for insulin resistance (HOMA2-IR) will be calculated.

Blood and urine samples will also be collected to measure pro-inflammatory/inflammatory, diabetic, obesity, and fibrotic markers. These include Interferon gamma (IFNγ), cystatin C, interleukin (IL)-6, IL-8, monocyte chemoattractant protein-1 (MCP-1), tumour necrosis factor alpha (TNFα), IL-10, vascular endothelial growth factor (VEGF), adiponectin, and glucagon in blood and Albumin/creatinine ratio (ACR) and MCP-1 in urine.

Additional pro-inflammatory/inflammatory, fibrosis, diabetic and obesity biomarkers in blood (plasma) and urine will be measured for exploratory analysis. The biomarkers to be analysed will include but are not limited to: free fatty acid (FFA), cholecystokinin (CCK), leptin, adipsin, ghrelin, resistin, visfatin, c-peptide, glucagon-like peptide 1 (GLP-1), plasminogen activator inhibitor 1 (PAI-1), gastric inhibitory peptide (GIP), alpha-1 antitrypsin (AAT), connective tissue growth factor (CTGF), intercellular adhesion molecule (ICAM-1), fibrinogen, and nitric oxide (NO) (all in plasma).

The above biomarkers will be analysed by the study site’s local research laboratory and partners as well as ProMetic BioSciences Inc.

#### Assessment of liver fibrosis

Transient Elastography (TE) is one of several methods, including liver MRI and the enhanced liver fibrosis (ELF) test, to measure the presence of liver fibrosis and response to PBI-4050. TE is a non-invasive technique to assess liver stiffness by measuring the velocity of sheer waves through the liver [26–28]. TE is performed using a Fibroscan (Echosense, France) with an M-probe (3.5 Hz frequency) or XL-probe (2.5 Hz frequency). All assessments are performed in the dorsal decubitus position with the ultrasound probe tip overlying the right lobe of liver [29].

MRI of the liver will be used to quantify liver fibrosis and response to PBI-4050. Using a 1.5 T (Siemens Avanto) scanner, the sequences acquired include 1) a central slice Shortened Modified Look-Locker Inversion recovery T1 sequence that times the longitudinal relaxation of each imaged voxel; 2) a multi-slice Spin Echo Imaging Inversion Recovery T1 mapping sequence; 3) a central slice high resolution T2 sequence that times the transverse relaxation of each imaged voxel; and 4) a central slice Dixon sequence that is used to separate water and fat.

An Enhanced Liver Fibrosis (ELF) test will be performed. The ELF test consists of three serum biomarkers and correlates with levels of liver fibrosis [30].

#### Assessment of cardiac fibrosis

Cardiac MRI will be used to quantify cardiac fibrosis and response to PBI-4050. Using a 1.5 T (Siemens Avanto) scanner left and right ventricular volumes, mass and function will be acquired using standard protocols. T1 mapping will be performed before and after gadolinium contrast using a Modified Look Locker Inversion recovery sequence employing a 5 s(3 s)3 s sampling protocol (average breath hold 10–15 s) to quantify diffuse myocardial fibrosis. Late gadolinium enhancement imaging will be performed 7–10 min after 0.15 mmol/Kg of gadolinium contrast bolus (Gadovist Bayer Health Care) to quantify extent of coarse replacement fibrosis [31].

#### Assessment of adipose tissue

Adipose tissue samples will be used to quantify the effects of PBI-4050 on fibrosis in adipocytes. Subcutaneous adipose tissue from ALMS subjects is to be characterised using histo-morphometry and immunohistochemistry staining. The biopsies are collected from the abdomen on the lateral side of the umbilicus on the abdominal wall. After administration of local anaesthetic (1% lignocaine), a 1-cm subcutaneous incision is made with a scalpel and the removed sample of adipose tissue is immediately preserved in 2 parts in formalin and RNAlater® Stabilization Solution (Thermofisher scientific). The samples stored in formalin are then embedded in paraffin to make formalin-fixed paraffin-embedded (FFPE) blocks. The FFPE blocks are cut and stained with haematoxylin and eosin and picrosirius-red staining for quantification of adipose tissue fibrosis. Adipocyte size is measured by means of light microscopy. Immunohistochemistry with macrophage, T-cell and collagen markers will be performed to quantify inflammatory activity and collagen deposition. Global gene expression in adipocytes will be measured. Adipose tissue stored in RNAlater is prepared using RNeasy Lipid Tissue (QIAGEN) and next generation gene sequencing performed to access expression of pro-fibrotic genes.

Adipose tissue microdialysis is used to sample interstitial fluid intermediary metabolites in the form of glycerol, glucose, pyruvate and lactate, which act as a marker of adipose tissue lipolysis. A microdialysis catheter (CMA microdialysis [CMA 106/107], Sweden) is inserted approximately 10 cm lateral to and at the level of the umbilicus via a subcutaneous incision after local anaesthetic (1% lignocaine). Samples are collected every 30 min into micro-vials (0.3 μL/min) until the end of the hyperinsulinaemic-euglycaemic glucose clamp.

#### Assessment of insulin secretion and resistance

The hyperinsulinaemic-euglycaemic glucose clamp is considered the gold standard for quantifying insulin secretion and resistance and will be used to monitor the effects of PBI-4050 on insulin resistance in ALMS [32]. In effect, blood glucose lowering effects of insulin are antagonised by glucose infused at a variable rate to keep blood glucose clamped at a specific fasting level [33].

Subjects are required to fast overnight before starting the hyperinsulinaemic-euglycaemic clamp at 0900 am the following morning. Fasting glucose is recorded and subjects are then clamped at that level. Subjects are given a bolus (2 mg/kg) of D-Glucose (U-13C6) (Cambridge Isotope Laboratories, UK) before a continuous infusion of D-glucose (20 μg/kg/min) for the next 4 h. An infusion of potassium palmitate 2, 2 D2 (Cambridge Isotope Laboratories, UK) in 5% albumin (0.03 μmol/kg/min) is given at the same time and continues for next 4 h until the end of procedure. During the first 2 h (basal phase), blood glucose is checked every 15 min and additional bloods are taken at time points 0, 90, 105, and 120 min for measurement of insulin, free fatty acid and stable isotopes for the assessment of whole body basal glucose turnover. Samples taken at time points 0, 90, 105, 120, 210, 225 & 240 will also be analysed with another reference laboratory method and clamp quality for these paired samples reported according to Benesch et al., 2015.

Insulin (Actrapid; Novo Nordisk, Copenhagen, Denmark) infusion is then started at 120 min at a rate of 40 mU/m^2^/min. Four minutes later, a 20% dextrose solution with [^13^C]-glucose (enriched to 4%) is commenced at time point 124 min. During the insulin-stimulated state period, blood sugars are checked every 5 min to allow for dextrose infusion rate adjustments to maintain fasting glycaemic levels. At time points 210, 225 and 240 min, steady state blood samples are collected. The rates of endogenous glucose production, glucose disposal and lipolysis (Rd - rate of disposal of palmitate) are calculated.

### Discontinuation or withdrawal of study subjects

PBI-4050 will be permanently discontinued for the following reasons:

- Subject’s withdrawal of consent;
- An AE, which in the opinion of the investigator warrants discontinuation of treatment;
- Pregnancy;
- Death;
- Substantial protocol deviation;
- Investigator’s decision;
- Sponsor’s decision;
- Subject starting a prohibited therapy (see Section 5.6);
- aspartate transaminase or alanine transaminase rises to > 5 × upper limit of normal;
- A drop in heart rate from baseline by 15 beats per minute that is associated with clinically relevant cardiovascular symptoms as determined by the investigator;
- A drop in systolic blood pressure by 20 mmHg or diastolic blood pressure by 15 mmHg from baseline that is associated with clinically relevant cardiovascular symptoms as determined by the investigator.

Subjects who discontinue study treatment but have not withdrawn consent will be asked to attend the appropriate End-of-Treatment and End-of-Study visits, depending upon whether they withdraw before or after Week 24. Subjects who withdraw consent will not have further study-related information collected for the clinical trial database, with the exception of follow-up on on-going AEs or any new AEs within the safety follow-up period. Subjects who withdraw due to AEs will be followed until the event has resolved or stabilized.

In the case of any substantial deviation from the protocol, the investigator and sponsor will decide on the further participation of the subject in this study, having discussed all relevant aspects. The sponsor may discontinue the study in its entirety before its completion for reasonable cause, provided that written notice to the investigator, regulatory authorities and independent ethics committee (EC) is submitted in reasonable time in advance of the intended termination.

If subjects decide to enter the ALMS rollover study (Study PBI-4050-CT-9-10) after completing the 36- or 48-week extension period, then they will sign an informed consent for that study and stop participation in the current study.

### Pharmacovigilance and data monitoring

All SAEs will be reported to pharmacovigilance/safety personnel within 24 h after the investigator becomes aware of the event. All relevant information about any suspected unexpected serious adverse reaction (SUSAR) occurring during the course of a clinical trial and if fatal or life-threatening will be reported as soon as possible and in any event not later than 7 days to the competent authority. All SUSARs occurring in the interventional study will be entered into a European database established in accordance Directive 2001/20/EC and reported to the relevant ECs. A Data Safety Monitoring Board will review individual subject safety data in an on-going fashion. An unaffiliated clinical research associate will monitor the data collected throughout the study, thus providing quality control (QC) of the study and QC of the database and data management.

### Sample size and statistical analysis

This study is purely exploratory and aims to evaluate safety and tolerability of PBI-4050 and examine its effects on biomarkers. The study will collect data to derive descriptive statistics on secondary endpoints for future studies and no statistical hypothesis will be tested. A minimum of 8 subjects is deemed necessary in this part of the study. The data will be summarized using mean, standard deviation, median, and range. Demographic (sex, age, race, ethnicity, waist circumference, height, weight, and BMI) and baseline characteristics will be presented descriptively at baseline. Categorical variables will be reported as numbers and percentages. Paired t-test or where appropriate the non-parametric equivalent (Wilcoxon signed-rank test) will be used to compare variables before and after intervention. The Shapiro-Wilk test will be used to test the normality of the distribution. In the event that the normality assumption fails at the 0.10 alpha level of significance, the Wilcoxon Signed-Rank test will be used instead. Absolute values and changes from baseline in safety laboratory tests will be summarized by visit over time and presented in shift tables. To evaluate the shift over time, a mixed model comparing the slope before baseline (historical data) to the slope after baseline (on-treatment data) will be used. For comparing results of repeated samples, for example adipose tissue microdialysis where there are multiple time points during the same test, repeated measures analysis of variance will be used. All analysis will be conducted using SPSS statistical software. The significance level for this study is set at *p* < 0.05. All statistical analyses will be made using observed data. No imputation will be made.

## Discussion

ALMS is a rare genetic disease characterised by multiorgan dysfunction and progressive fibrosis, resulting in reduced life expectancy [1]. Given its rarity and complexity, clinical trials in this disease are challenging. The national centre for ALMS in Birmingham UK was selected as the centre able to deliver this study in 18 subjects. A single-arm, open-label design was selected to maximise subject exposure and increase the likelihood of achieving the chosen study endpoints.

Study PBI-4050-ATX-9-05 will initially evaluate the safety and tolerability of PBI-4050 over a 24-week period, during which therapeutic efficacy in terms of modification of the metabolic syndrome, liver and cardiac fibrosis and pro-inflammatory and pro-fibrotic state will be assessed, followed by an exploratory phase in those subjects who are tolerating therapy. Because of the complexity of the ALMS phenotype, a variety of investigations are incorporated into the study. This study is not intended as a full assessment of the safety and efficacy of PBI-4050. Rather, this study will provide valuable safety and biomarker evidence to support further development of PBI-4050 for the treatment of ALMS, a disease with severe morbidity and early mortality in which PBI-4050 has the potential to improve these outcomes.

### Trial status

Recruitment to Study PBI-4050-ATX-9-05 began in February 2016. The study has recruited 12 subjects in the main study and 9 subjects in the extension phase. The study is currently recruiting in the UK and scheduled to end in 2018.

## Additional files

Additional file 1: Schedule of Study Procedures (Main Study). Study Visits Initial 24 Weeks. Study Visits during the Initial 24 Weeks of Treatment. The schedule of study procedures for the enrolment, intervention, and assessments for participants is presented in detail in Additional file 1 (main study) and Additional file 2 (extension period). (PDF 218 kb)

Additional file 2: Schedule of Study Procedures (Extension Period). Study Visits during Extension Period. Study Visits During the 36- or 48-Week Extension Period. The schedule of study procedures for the enrolment, intervention, and assessments for participants is presented in detail in Additional file 1 (main study) and Additional file 2 (extension period). (PDF 207 kb)

Additional file 3: Exclusion criteria. This file list the exclusion criteria for the study. Subjects will not enter the study if any of the exclusion criteria listed in Additional file 3 is fulfilled. (PDF 281 kb)

## Abbreviations

AE: Adverse events
ALMS: Alström syndrome
ELF: Enhanced Liver Fibrosis
FPG: Fasting plasma glucose
HbA1c: Glycated haemoglobin
IMP: Investigational Medicinal Product
MRI: Magnetic resonance imaging
NT-pro-BNP: N-terminal pro-brain natriuretic peptide
SAE: Serious adverse event
T2DM: Type 2 diabetes mellitus
TE: Transient elastography
