# Supplementary material for: Treatment with PBI-4050 in patients with Alström syndrome: study protocol for a phase 2, single-Centre, single-arm, open-label trial
Source: BMC Endocr Disord. 2018 Nov 26;18:88. doi: 10.1186/s12902-018-0315-6 (PMC6258144; doi:10.1186/s12902-018-0315-6)
Supplement: Supplementary file 1 — Schedule of Study Procedures (Main Study). Study Visits Initial 24 Weeks. Study Visits during the Initial 24 Weeks of Treatment. The schedule of study procedures for the enrolment, intervention, and assessments for participants is presented in detail in Additional file 1 (main study) and Additional file 2 (extension period). (PDF 218 kb) [file 12902_2018_315_MOESM1_ESM.pdf]

Supplementary File 1: Study Visits during the Initial 24 Weeks of Treatment

Visit	Screening	Wk 1	Wk 4	Wk 8	Wk 12	Wk 16	Wk 20	Wk 24 / EoT	EoS
Informed consent	X							X	
Demography	X								
Medical history	X								
Medication history	X								
Eligibility review	X								
Physical examination	X	X			X			X	X
Height	X								
Weight and waist circumference	X	X[b]	X	X	X	X	X	X	X
Vital signs	X	X[b]	X	X	X	X	X	X	X
Haematology	X	X[c]	X	X	X	X	X	X	X
Biochemistry	X	X[c]	X	X	X	X	X	X	X
Urinalysis	X	X[c]	X	X	X	X	X	X	X
Pregnancy test[d]	X	X[b]	X	X	X	X	X	X	X
12-lead ECG	X	X[b]			X			X	X
FibroScan		X[e]			X			X	
Liver MRI		X[e]						X	
Cardiac MRI		X[e]						X	
Hyperinsulinaemic-euglycaemic		X[e]						X	

Visit	Screening	Wk 1	Wk 4	Wk 8	Wk 12	Wk 16	Wk 20	Wk 24 / EoT	EoS
clamp and adipose tissue microdialysis									
Adipose tissue biopsies[f]		X[e]						X	
Adverse events	X	X	X	X	X	X	X	X	X
Concomitant medications	X	X	X	X	X	X	X	X	X
Metabolic syndrome parameters (FPG and fasting insulin)		X			X			X	
Metabolic syndrome parameter (HbA1c)	X	X	X	X	X	X	X	X	X
Biomarkers[g]		X	X	X	X	X	X	X	
Diary dispensation / review[h]		X	X	X	X	X	X	X	X
IMP accountability and compliance			X	X	X	X	X	X	
IMP administration (daily at home)[i]		X	X	X	X	X	X	X	
Fasting BG (daily at home)[j]		X	X	X	X	X	X	X	X[k]
4-point glucose profile (weekly at home)[j,l]			X	X	X	X	X	X	X[k]

BG=blood glucose, ECG=electrocardiogram, EoT=End of Treatment, EoS=End of Study, FPG=fasting plasma glucose, HbA1c=haemogloblin A1c, IMP=Investigational Medicinal Product, MRI=magnetic resonance imaging.

[a] Assessments at the Week 1 baseline and Week 24/EoT visits may be completed over 2 days if necessary, as scheduled by the Investigator.

[b] Physical examination, weight and waist circumference, vital signs, ECG, and pregnancy test do not need to be repeated if they have been performed within 2 days before the Week 1 baseline visit.

[c] Baseline haematology, biochemistry, and urinalysis do not need to be repeated if they have been performed within 7 days before the Week 1 baseline visit.

[d] Women with childbearing potential only. Either serum or urine pregnancy test is permitted.

[e] Baseline FibroScan, MRIs, clamp test and adipose tissue microdialysis, and adipose tissue biopsy may be performed within the screening period before the Week 1 baseline visit.

[f] Optional procedure.

[g] Blood and urine samples are obtained to measure pro-inflammatory markers, diabetic, obesity, and fibrotic markers and cardiac function marker as described in the protocol.

[h] Completed diary is collected at each visit and new diary is dispensed. Study personnel will also question the subjects and review diary records during telephone contacts.

[i] The IMP should be taken orally (four capsules of 200 mg PBI-4050) by subjects once daily one hour before or two hours after a meal, preferably at the same time every day. Enough supply will be provided at each visit. The first dose is taken on Day 1 either at home or at the study site.

[j] Fasting BG and weekly 4-point glucose profile are performed at home by subjects or caregivers from Week 1 through EoS. Results should be recorded in the diary and will be reviewed and collected by study personnel at each visit. Fasting BG is not measured at home on study visit days.

[k] Fasting BG and weekly 4-point profile recorded in the diary for the period between Week 24/EoT and EoS are collected at the EoS visit.

[l] Weekly 4-point glucose profile is required only for subjects who are taking insulin.

[m] Subjects who enter the EP at Week 24 will sign informed consent and continue ongoing study medication without any break in treatment.
