# Supplementary material for: Treatment with PBI-4050 in patients with Alström syndrome: study protocol for a phase 2, single-Centre, single-arm, open-label trial
Source: BMC Endocr Disord. 2018 Nov 26;18:88. doi: 10.1186/s12902-018-0315-6 (PMC6258144; doi:10.1186/s12902-018-0315-6)
Supplement: Supplementary file 2 — Schedule of Study Procedures (Extension Period). Study Visits during Extension Period. Study Visits During the 36- or 48-Week Extension Period. The schedule of study procedures for the enrolment, intervention, and assessments for participants is presented in detail in Additional file 1 (main study) and Additional file 2 (extension period). (PDF 207 kb) [file 12902_2018_315_MOESM2_ESM.pdf]

Supplementary File 1: Study Visits During the 36- or 48-Week Extension Period

**Visit**	**Start of Extension Study[a]**	**EP Week 12**	**EP Week 24**	**EP Week 36[j] / EoT**	**EP Week 48[k] / EoT**	**EoS**
Informed consent	X					
Demography						
Medical history						
Medication history						
Eligibility review	X[b]					
Physical examination	X[b]	X	X	X	X	X
Height						
Weight and waist circumference	X	X	X	X	X	X
Vital signs	X	X	X	X	X	X
Haematology	X	X	X	X	X	X
Biochemistry	X	X	X	X	X	X
Urinalysis	X	X	X	X	X	X
Pregnancy test[c]	X	X	X	X	X	X
12-lead ECG	X	X	X	X	X	X
FibroScan		X	X	X	X	
Liver MRI				X		
Cardiac MRI				X		

**Visit**	**Start of Extension Study**[a]	**EP Week 12**	**EP Week 24**	**EP Week 36**[j] **/ EoT**	**EP Week 48**[k] **/ EoT**	**EoS**
Adverse events	X	X	X	X	X	X
Concomitant medications	X	X	X	X	X	X
Metabolic syndrome parameters (FPG and fasting insulin)		X	X	X	X	
Metabolic syndrome parameter (HbA1c)	X	X	X	X	X	X
Biomarkers[d]		X	X	X	X	
Diary dispensation / review[e]	X	X	X	X	X	X
IMP accountability and compliance		X	X	X	X	
IMP administration (daily at home)[f]	X	X	X	X	X	
Fasting BG (daily at home)[g]		X	X	X	X	X[h]
4-point glucose profile (weekly at home)[g,i]		X	X	X	X	X[h]

BG=blood glucose, ECG=electrocardiogram, EP=Extension Period; EoT=End of Treatment, EoS=End of Study, FPG=fasting plasma glucose, HbA1c=haemogloblin A1c, IMP=Investigational Medicinal Product, MRI=magnetic resonance imaging.

[a] Subjects who have already stopped study medication (after Week 24) and have not completed an EoS visit will undergo a Start of Extension Study visit. Subjects who have already stopped study medication and completed a EoS visit will have to be re-assessed for study eligibility and will undergo a Start of Extension Study visit. Subjects who enter the extension period at the Week 24 visit will not have a Start of Extension Study visit, they will be dispensed IMP at the Week 24 visit and the Week 24 visit will serve as EP Day 0.

[b] Eligibility review and physical examination will only be performed in subjects who have already stopped study medication (after Week 24) and completed a EoS visit

[c] Women with childbearing potential only. Either serum or urine pregnancy test is permitted.

[d] Blood and urine samples are obtained to measure pro-inflammatory markers, diabetic, obesity, and fibrotic markers and cardiac function marker as described in the protocol.

[e] Completed diary is collected at each visit and new diary is dispensed. Study personnel will also question the subjects and review diary records during telephone contacts.

[f] The IMP should be taken orally (four capsules of 200 mg PBI-4050) by subjects once daily one hour before or two hours after a meal, preferably at the same time every day. Enough supply will be provided at each visit. The first dose is taken from Week 24/Start of Extension Study visit either at home or at the study site.

[g] Fasting BG and weekly 4-point glucose profile are performed at home by subjects or caregivers from Week 24/Start of Extension Study visit through EoS. Results should be recorded in the diary and will be reviewed and collected by study personnel at each visit. Fasting BG is not measured at home on study visit days.

[h] Fasting BG and weekly 4-point profile recorded in the diary for the period between EP Week 36 or 48/EoT and EoS are collected at the EoS visit.

[i] Weekly 4-point glucose profile is required only for subjects who are taking insulin.

[j] At the completion of the EP Week 36 visit, subjects will be allowed to enrol in Alström Rollover Study PBI-4050-CT-9-10 if it is open for enrolment (if enrolment is open, then the EP Week 36 visit will be the EoT visit) and continue ongoing study medication without any break in treatment. If subjects decide to enter Alström Rollover Study PBI-4050-CT-9-10, they will sign an informed consent for that study and stop participation in the current study. If Alström Rollover Study PBI-4050-CT-9-10 is not open for enrolment, they will continue in the current study through the EoS visit.

[k] At the completion of the EP Week 48 visit, subjects will be allowed to enrol in Alström Rollover Study PBI-4050-CT-9-10 and continue ongoing study medication without any break in treatment. If subjects decide to enter Alström Rollover Study PBI-4050-CT-9-10, they will sign an informed consent for that study and stop participation in the current study. If subjects decide to not enter Alström Rollover Study PBI-4050-CT-9-10, they will continue in the current study through the EoS visit.
