# Supplementary material for: Treatment with PBI-4050 in patients with Alström syndrome: study protocol for a phase 2, single-Centre, single-arm, open-label trial
Source: BMC Endocr Disord. 2018 Nov 26;18:88. doi: 10.1186/s12902-018-0315-6 (PMC6258144; doi:10.1186/s12902-018-0315-6)
Supplement: Supplementary file 3 — Exclusion criteria. This file list the exclusion criteria for the study. Subjects will not enter the study if any of the exclusion criteria listed in Additional file 3 is fulfilled. (PDF 281 kb) [file 12902_2018_315_MOESM3_ESM.pdf]

Supplementary File 1: Exclusion criteria

**Exclusion Criteria**
1. Subject has recent or on-going infection requiring systemic treatment with an anti-infective agent within 30 days before screening.
2. Subject has had at least two documented episodes of severe hypoglycaemia within 12 months before screening. Severe hypoglycaemia is defined as an episode of confirmed hypoglycaemia (glucose < 3.0 mmol/L) that required parenteral treatment with intramuscular injection of glucagon or intravenous injection of dextrose, an episode of confirmed hypoglycaemia that did not require parenteral treatment but involved severe neuroglycopenic symptoms, or an episode of unconfirmed hypoglycaemia that resulted in seizure or coma.
3. Subject has uncontrolled hypertension with 170/100 mm Hg as determined at screening.
4. Subject has alanine transaminase (ALT) or aspartate transaminase (AST) level ≥ 5 × upper limit of normal (ULN) at screening.
5. Subject is currently using weight loss medications at screening. Subjects may be re-screened after stopping the weight loss medication for a period of at least 5 half-lives.
6. Subject has a history of chronic alcohol or other substance abuse as determined at screening.
7. Woman who is pregnant, breast-feeding, or planning a pregnancy during the course of the study as determined at screening.
8. Subject has any condition that, in the investigator’s opinion, is likely to interfere with study conduct and compliance.
9. Subject has a history of an allergic reaction to PBI-4050 or any of its excipients.
